# Algorithmic Approaches for Assessing Irreversibility in Time Series: Review and Comparison

**DOI:** 10.3390/e23111474

**Published:** 2021-11-08

**Authors:** Massimiliano Zanin, David Papo

**Affiliations:** 1 Instituto de Física Interdisciplinar y Sistemas Complejos (CSIC-UIB), Campus Universitat de les Illes Balears, E-07122 Palma de Mallorca, Spain; 2 Department of Neuroscience and Rehabilitation, Section of Physiology, University of Ferrara, 44121 Ferrara, Italy; david.papo@unife.it; 3 Fondazione Istituto Italiano di Tecnologia, 44121 Ferrara, Italy

**Keywords:** irreversibility, time-reversal symmetry, nonlinearity

## Abstract

The assessment of time irreversibility, i.e., of the lack of invariance of the statistical properties of a system under the operation of time reversal, is a topic steadily gaining attention within the research community. Irreversible dynamics have been found in many real-world systems, with alterations being connected to, for instance, pathologies in the human brain, heart and gait, or to inefficiencies in financial markets. Assessing irreversibility in time series is not an easy task, due to its many aetiologies and to the different ways it manifests in data. It is thus not surprising that several numerical methods have been proposed in the last decades, based on different principles and with different applications in mind. In this contribution we review the most important algorithmic solutions that have been proposed to test the irreversibility of time series, their underlying hypotheses, computational and practical limitations, and their comparative performance. We further provide an open-source software library that includes all tests here considered. As a final point, we show that “one size does not fit all”, as tests yield complementary, and sometimes conflicting views to the problem; and discuss some future research avenues.

## 1. Introduction

For a viewer watching the movie of an ideal pendulum undergoing no friction it is impossible to tell whether the movie is played forward or in reverse, the only differences between these two motions being the pendulum’s initial position and speed. The system’s motion is then said to have time-reversal symmetry. More formally, time reversal symmetry is the symmetry of a physical law or a realisation of a physical phenomenon under the time-reversal transformation t↦−t.

There are at least two somehow dual approaches to time-reversal symmetry. In the first approach, observed phenomena are thought of as a realisation of a stochastic dynamical process, and the goal is to try to understand what information the time-reversal symmetry and its breakdown provide on the statistical properties of these processes [1,2,3,4]. In a statistical sense, time-reversal symmetry quantifies the extent to which it is possible to discern a preferred time direction of a time ordered realisation of some stationary stochastic process $\ldots ,\;X_{-2},\;X_{-1},\;X_{0},\;X_{1},\;X_{2},\;\ldots$, i.e., the extent to which it can be distinguished from the time-reversed sequence $\ldots ,\;X_{2},\;X_{1},\;X_{0},\;X_{-1},\;X_{-2},\;\ldots$ [5].

Once time-reversal symmetry is treated as a property of a given time series, it can be shown that reversibility implies stationarity [3] and, furthermore, that linear Gaussian random processes and static non-linear transformations of such processes are reversible. Conversely, the presence of time irreversibility rules out Gaussian linear processes or linear autoregressive moving average (ARMA) models as possible generating dynamics, implying instead non-linear dynamics or (linear or non-linear) non-Gaussian [1,2,3,4]. Time-reversal symmetry can be due to the presence of dissipative forces which are reflected by long-range correlations and nonlinear dependences, and, therefore, in turn affecting the degree of time series predictability. The presence of time irreversibility implies nonlinear dynamics, non-Gaussian (linear or nonlinear), or linear ARMA models as possible generative processes, ruling out Gaussian linear models, such processes and their static nonlinear transformations being reversible. Memory acts as a hidden dissipative external force [6], while the presence of noise results in loss of perceived temporal asymmetry of a given process [7,8].

The second approach to time-reversal symmetry focuses on the association of this symmetry with deep properties of physical and biological systems characterising their intrinsic modus operandi. An essential characteristic of irreversible thermodynamic processes is the breakdown of the time-reversal symmetry of the microscopic dynamics [9]. Moreover, broken time-reversal symmetry is the hallmark of systems operating away from equilibrium [10]. Such systems use part of their free energy budget to perform work or store it in alternative forms, dissipating the rest as heat in the environment, and this is associated with an irreversible increase in entropy of the environment, in agreement with the second law of thermodynamics.

The rate of entropy production, or dissipation, is associated with broken time-reversal symmetry of the associated dynamics, its rate constituting a measure of statistical irreversibility of non-equilibrium processes [7,11,12,13]. When dissipation is large, time asymmetry becomes conspicuous, reflecting the price in entropy lost to dissipation. Hence the idea that time-reversal symmetry can be used not only as an indicator of whether a system is at equilibrium or not, but also as a quantifier of its distance from such a condition [14].

The relationship between irreversibility of thermodynamic processes, the breakdown of time-reversal symmetry expressing it, and entropy production in systems operating away from equilibrium can be investigated in various ways.

Time-reversal symmetry breaking may be signalled by directed flows or currents. Irreversibility may therefore be detected by highlighting the existence of such flows or fluxes [10]. This can be done by using different but related symmetries of the system and measuring their breakdown. One possible strategy makes use of the fact that equilibrium fluctuations obey detailed balance of the probability fluxes: the net current between any pair of states vanishes at long enough times, i.e., given two states $w_{1}$ and $w_{2}$ and a transition rate W(·) following condition holds:

(1) $$\rho \left(w_{1}\right)W\left(w_{1}\to w_{2}\right)=\rho \left(w_{2}\right)W\left(w_{2}\to w_{1}\right),$$

where ρ(·) is the equilibrium probability distribution. Thus, in particular, every forward microstate trajectory must have the same probability to occur as its time-reverse counterpart. Non-equilbrium steady states can break detailed balance, with the formation of flux loops [10,15]. This condition can be understood in terms of the symmetry property of the probability distributions $P\left(w_{t}\right)=P\left(Iw_{t}\right)$ of a trajectory $w_{t}=\left(w_{1},w_{2},\ldots ,w_{t}\right)$ of length *t* and its time-reversed one, where *I* is the time reversal operator. An important result is that the ratio between these two quantities, which quantifies the distance between the forward and backward path in the appropriate space, provides a estimate of the thermodynamic entropy production $(d_{i}S)/dt$ [9,16,17,18]. The ratio $d_{i}S/dt=\ln\left[P\left(w_{t}\right)/P\left(Iw_{t}\right)\right]$ is strictly positive for non-equilibrium systems and vanishes only if detailed balance is satisfied. This ratio can also be interpreted in terms of the Kullback-Leibler distance $D(\rho ||\tilde{\rho }$) between the space phase densities ρ and $\tilde{\rho }$ respectively of the forward and backward path. For non-equilibrium steady state systems, this relation can be expressed in terms of Kolmogorov-Sinai entropy of the forward and backward paths, respectively *h* and $h^{R}$ [11,16]:

(2) $$\frac{d_{i}S}{dt}=k_{B}\left(h^{R}-h\right).$$

This relation indicates that in non-equilibrium steady states, the typical paths are more ordered in time than the corresponding time-reversed paths, pointing to a fundamental relationship between system’s dynamics and its thermodynamics [16]. It is also directly related to Landauer’s principle, which quantifies the minimal dissipation associated with information erasure [19,20]. Remarkably, the left-hand side is a purely physical macroscopic quantity, whereas the right-hand side is a statistical magnitude associated with microscopic fluctuations, which does not depend on the generating mechanism [21].

Instead of studying the ratio of the two aforementioned quantities, which quantifies the distance between the forward and backward path in the appropriate space providing an estimate of the entropy production $(d_{i}S)/dt$ by means of Kolmogorov-Sinai entropy in the previous equation, one may employ the entropy change ΔS under time reversal [22] of the entropy *S* in natural time analysis [23,24,25,26], which in general does satisfy the requirement to identify time-reversal asymmetry. The advantage of using ΔS is that the study of its fluctuations can identify when a system approaches a dynamic phase transition, as for example the occurrence of a large earthquake [22,27].

A further way to quantify irreversibility and therefore dissipation can be found by looking at a related symmetry and its breakdown, i.e., by experimentally assessing [28,29] violations of the fluctuation-dissipation relations, connecting equilbrium system’s spontaneous fluctuations, response to exogenous perturbations and temperature [30,31]. Violations of the fluctuation-dissipation relations reflect the breakdown of detailed balance and the extent of the violation is related to the system’s rate of dissipation [32]. The departure from equilibrium is often studied by introducing effective temperatures [33]. However, in addition to correlations of the unperturbed system, this approach also requires active measuring of the system’s response properties to particular fields that leave the system sufficiently close to equilibrium. Moreover, there is no direct relation between effective temperatures and time-reversal symmetry breaking.

More generally, non-equilibrium systems obey fluctuation relations ruling the fluctuating amounts of work or entropy produced during far from equilibrium processes, which hold for any stationary process, independently of the dynamics [34,35,36,37,38]. These relations describe the behaviour of a system driven from equilibrium by an external perturbation. Contrary to linear response theory, these expressions are exact irrespective of the perturbation strength, or of how far from equilibrium the system has been driven to. In particular, the relation

(3) $$P\left(-\Sigma_{t}\right)∼P\left(\Sigma_{t}\right)e^{-\Sigma_{t}}$$

where $\Sigma_{t}=d_{i}S/dt$ is the entropy production probability for a finite non-equilibrium system flowing against the direction mandated by the second law of thermodynamics. This fluctuation relation can be seen as a symmetry property of the entropy current’s large deviation function resulting from driving the system into a non-equilibrium steady state [35,39].

Importantly, for a large class of systems, thermodynamic quantities like heat, work and entropy can be defined at the level of single stochastic trajectories [40,41], bridging the gap between the two seemingly separate approaches to time-reversal symmetry sketched above. From these can be derived not only fluctuation relations, but also statistical bounds, known as thermodynamic uncertainty relations [42,43], on the precision [42], rate [44] and speed of state transformation [45] of the underlying process [46].

### 1.1. Applications

These two approaches to time-reversal symmetry have been applied to the experimental study of very diverse physical, economical and biological systems.

One of the earliest applications of time-reversal symmetry analysis addressed the long-standing question in hydrodynamic turbulence of the nature of the cascade describing its emergence; it was shown that it constitutes a genuine dynamical process involving a time arrow rather than an instantaneous spatial structure, viscosity turning out to be key to the symmetry breakdown [47]. A similar finding was associated with the avalanche structure of financial time series. In particular, the two-point correlation functions of intraday return volatility across time scales was shown to be characterised by a causal information cascade from large to fine scales [48]. More generally, time reversal symmetry metrics have been used to characterize economic and financial time series [8,49,50,51,52]. In the domain of finance, various empirical studies have been undertaken to test the so-called Efficient Market Hypothesis [53], whereby a market is said to be efficient if asset prices incorporate all publicly available information, so that no single agent can on average outperform the market using such information. Thus, efficiency is related to the amount of information available to predict future market prices, with lower efficiency corresponding to higher residual predictive information in the past sequence of stock prices [54]. Financial time series have been found to be time irreversible [8,49,55,56], the degree of asymmetry varying greatly from stock to stock [51]. Furthermore, time-reversal symmetry breaking allows ranking a given market’s quality, mature ones turning out to be more time-symmetric than emerging ones [57]. Musical pieces ranging from Renaissance to the early Modern period have also been found to be characterised by time-reversal symmetry, associated with the presence of strong nonlinearities, indicating a structure richer than that of previously reported pink noise [58].

Insofar as time-reversal symmetry reflects the way a system produces entropy, and hence the extent to which it operates away from equilibrium, time irreversibility becomes a good descriptor of biological systems at all scales. Irreversibility, and the associated entropy production, are indeed fundamental elements for understanding life development and maintenance [59,60]. At microscopic scales, time-reversal symmetry breaking has been found in hair-cell bundle fluctuations. Hair cells in the inner ear transduce sound-evoked mechanical vibrations of their hair bundle into electrical signals, which are then sent to the brain. It was first shown that their spontaneous fluctuations violate fluctuation-dissipation theorem, indicating that such fluctuations are active ones [28] and are therefore characterised by probability phase spaces fluxes and entropy production. Recently, it was further shown that such dynamics are characterised by time-reversal symmetry [61].

At meso- and macroscopic scales, time reversal asymmetry has been used to characterise healthy and pathological activity of various biological systems. One of the most studied such systems is the human heart [62,63,64,65,66,67,68,69]. In particular, it was found that heart period fluctuations are characterised by time reversal asymmetry stemming from faster heart decelerations than accelerations, which result in period variability with steeper upward than downward side. Furthermore, a portion of heart period variability is due to the cardiac arm of the baroreflex. This reflex also shows asymmetry, as it is influenced by the sign of arterial pressure variations, which more efficiently compensates systolic rises than drops [70]. At even more coarse-grained scales, time-reversal symmetry has been used to characterise the dynamics of human limbs; for instance, it has been used to classify hand tremors [71], and to analyse gait, comparing healthy subjects and patients with Alzheimer’s dementia [72,73].

The time-reversal symmetry properties of brain activity have also attracted some attention [74,75,76,77,78,79]. Resting brain activity has been shown to be generically time irreversible, time-reversal symmetry being modulated by opening or closing eyes [78], phase of sleep [80], and altered in a pathology-specific way in psychiatric and neurological disease [78]. Temporal irreversibility appears to be the hallmark of wakeful consciousness [81]. Indices of time asymmetry seem to be altered at various scales in brain pathology and may therefore be used as diagnostic tools [82]. In particular, time symmetry appears to be altered in a disease-specific way in psychiatric and neurological pathologies including schizophrenia, Alzheimer’s and Parkinson’s diseases [78]. Moreover, in epilepsy, increased irreversibility has consistently been reported for ictal activity in both scalp and intracranially recorded electrical brain activity [75,79,83,84]. Importantly, surgical removal of brain areas with irreversible intracranial electroencephalography (iEEG) signals was associated with seizure-free postsurgical outcome [83].

### 1.2. Assessing Irreversibility in Real-World Time Series

Assessing time reversal symmetry is not an easy task. This is due not only to the many aetiologies of irreversibility, but also to the theoretical background within which irreversibility is studied and to the experimental evaluation of the properties each method strives to quantify. As a result, various methods have been proposed in the last decades, based on different principles, goals and applications. In the remainder, we illustrate some of these methods, comparing their performance in various contexts.

The aforementioned comparison will here be based on a large set of synthetic (Section 3.2) and real (Section 3.4) time series, the former ones created using well-known dynamical systems. Additionally, we here assume that the researcher has no prior information about the time series, including information about its (non-)stationarity. It is worth noting that a non-stationary time series is by definition irreversible, and that the classical approach to irreversibility has been to disregard such time series. On the contrary, we here include also non-stationary models (as, e.g., the Generalised Autoregressive Conditional Heteroskedasticity model [85]), with the aim of not just detecting this trivial irreversibility, but also of quantifying it (for a deeper discussion on this topic, see [86]).

We complement this analysis with an open-source software library, which integrates the main tests here discussed, and that has been the basis of all reported results-see Section 4. We invite the reader to check and use it, and send to us any suggestions for improvement.

We finally close this review with a discussion of the limitations of the methods hitherto proposed, and of the avenues for their further development.

## 2. Numerical Methods

### 2.1. The BDS Statistic

The BDS statistic was initially proposed by Broock, Dechert and Scheinkman in a working paper in 1988, then being published as [87] and extensively tested in [88]. This test was designed to check for the presence of low-dimensional chaos in economic and financial data, and hence the adequacy of fitting a linear model to a time series; the use of the BDS statistic for assessing time irreversibility was a later application.

Given a time series $\left\{x_{t}\right\}$ composed of *T* observations, the statistic is defined as:

(4) $$w_{d}\left(r,T\right)=\sqrt{T}\frac{C_{m}\left(r,T\right)-C_{1}{\left(r,T\right)}^{m}}{\sigma_{m}\left(r,T\right)},$$

where $C_{m}\left(r,T\right)$ is the sample correlation integral at embedding dimension *m* and scaling parameter *r*, and $\sigma_{m}\left(r,T\right)$ is the estimated standard deviation of the BDS statistic under the null hypothesis of independence. $w_{d}$ is distributed asymptotically as N(0,1) under the null hypothesis of independently and identically distributed data.

### 2.2. Ramsey and Rothman’s Time Reversibility Test

The second metric for detecting irreversibility from time series was proposed by James B. Ramsey and Philip Rothman in 1996 [49]. Given a zero mean and stationary time series $\left\{x_{t}\right\}$ composed of *T* observations, it is based on calculating the method-of-moments estimators of two sample bicovariances:

(5) $${\hat{B}}_{2,1}\left(k\right)={\left(T-k\right)}^{-1}\sum_{t=k+1}^{T}x_{t}^{2}x_{t-k},$$

and

(6) $${\hat{B}}_{1,2}\left(k\right)={\left(T-k\right)}^{-1}\sum_{t=k+1}^{T}x_{t}x_{t-k}^{2},$$

for various integer values of *k*. The estimator ${\hat{\gamma }}_{2,1}\left(k\right)={\hat{B}}_{2,1}\left(k\right)-{\hat{B}}_{1,2}\left(k\right)$ is then constructed, and under the null hypothesis that the time series is reversible, the value of ${\hat{\gamma }}_{2,1}\left(k\right)$ is expected to be zero for all *k*. As ${\hat{\gamma }}_{2,1}\left(k\right)$ is asymptotically normally distributed, a simple T-test can be performed to check whether ${\hat{B}}_{2,1}\left(k\right)$ and ${\hat{B}}_{1,2}\left(k\right)$ have identical average values. Through the use of Monte-Carlo simulations, the Ramsey and Rothman’s test (called Time Reversibility test in the original paper, and here shortened to Ramsey’s test for the sake of clarity) has been proved to have a higher power than the BDS test and excellent size properties [89].

### 2.3. The DFK Test

The DFK test, proposed by Daw, Finney and Kennel [90], is based on the symbolisation of the time series. This involves partitioning the data range and assigning a symbol to each time series’ value based on the region into which it falls. While this partitioning can be done in a variety of ways, the simplest one involves dividing the data range in *n* equiprobable regions. After all values are assigned to a partition, and hence transformed to “symbols”, groups of *L* of these are merged to create “words”. To illustrate, suppose the time series x={3,5,4,3,6,6}; for n=2, two ranges are created, corresponding respectively to the ranges x<4.5 and x>4.5, thus yielding a symbolised time series {1,2,1,1,2,2}; for L=3, triplets of consecutive symbols are joined together, yielding the sequence of words (121), (211), (112) and (122). As a final step, the probability of appearance of each word is calculated, both for the original and the time-reversal version of the time series, and the two probability distributions are compared using a $\chi^{2}$ test.

### 2.4. Permutation Patterns (Permp) Test

Over the past few years, multiple irreversibility tests based on the concept of permutation patterns have independently been proposed [52,84,91,92,93]. The underlying element is the symbolisation of a time series using permutation patterns, i.e., (usually short) patterns describing the order that has to be applied to the elements of a time series to sort them. To illustrate, the permutation pattern associated to values (8,4,5) would be (2,3,1), as the second element is the smallest one, followed by the third and the first. The frequencies of these patterns have proven to be useful descriptors of the underlying dynamics, as e.g., of the degree of complexity (or determinism *vs.* stochasticity) of time series [94,95,96].

Constructing an irreversibility test based on permutation patterns is easily done. Given a time series $X=\{x_{1},x_{2},\ldots ,x_{N}\}$ composed of *N* values, this is divided into N−δ+1 overlapping windows of length δ, such that the *i*-th window is defined as $w_{i}=\left\{x_{i},\ldots ,x_{i+\delta -1}\right\}$. Values composing each window are then sorted from smaller to larger, and the corresponding permutation pattern $\pi_{i}$ is extracted. One can further note that, in a reversible time series, the distribution of the permutation pattern probabilities p(π) should be the same in the original and time reversed time series. This similarity can be assessed through the Jensen-Shannon divergence, i.e., a measure of the similarity between two probability distributions [97]. Finally, a test based on surrogate time series can be performed to check the statistical significance of the difference between the two distributions, and eventually obtain a *p*-value [84].

It is worth noting that the previously described testing strategy, based on surrogate time series, is computationally very expensive; for instance, in order to check for *p*-values below 0.01, the irreversibility test has to be performed to at least $10^{2}$, but more commonly $>10^{4}$, surrogate time series. An alternative can be obtained by observing that permutation patterns pair under the operation of time reversal; for instance, pattern (1,2,3) becomes (3,2,1), and so forth. If the time series is reversible, those pairs of patterns should appear with approximately the same frequency, otherwise they can be used to fix a direction of the arrow of time. Irreversibility can then be detected through binomial tests applied to these pairs of patterns [52].

The various studies proposing a permutation pattern-based irreversibility measure based on permutation patterns [52,84,91,92,93] mainly differ in the statistics used to assess the significance of the results. While we here use the method proposed in [52], the reader should be aware of the available alternatives [84,92,93].

### 2.5. The Ternary Coding (TC) Test

A conceptually similar but simplified version of the previous permutation pattern test was proposed ten years earlier by Cammarota and Rogora [98], and is based on the analysis of the ternary coding (TC) of the differentiated time series. Specifically, let us start from a time series $\left\{x_{t}\right\}$ composed of *T* observations; from this, a differentiated time series $\left\{d_{t}\right\}$ is obtained, whose elements are defined as $d_{i}=x_{i+1}-x_{i}$. The elements of this latter series are then coded as follows:

(7) $$s_{t}=\left\{\begin{array}{cc} 1, & \mathrm{if}\;d_{t}>\alpha \\ -1, & \mathrm{if}\;d_{t}<-\alpha \\ 0, & \mathrm{otherwise}, \end{array}\right.$$

with α being a user-defined constant. Note that, in synthesis, elements of the original time series are transformed into +1 and −1, respectively representing large increases and decreases; also note that 0 is not used in subsequent analyses. An irreversibility test can be constructed by noting that a sequence of consecutive +1 values of $s_{t}$ will transform into a sequence of −1 values under a time reversal operation-0 values will map into themselves, and this is why they are not taken into account. As with permutation patterns, a time series will be reversible if and only if the number of +1 and −1 sequences are not different in a statistical significant way. Given a sequence length, that in the case of ref. [98] was three elements, one can then count the number of sequences composed of only +1 values (denoted as $N^{+}$) and of only −1 values (denoted as $N^{-}$). A simple binomial test could be performed at this stage, i.e., to check whether the probability associated to $N^{+}$ is statistically different from the one associated to $N^{-}$. Cammarota and Rogora instead opted for another possibility, and specifically split the original time series into long non-overlapping segments (in the original work, these segments were of length 1000); calculate $N^{+}\left(i\right)$ and $N^{-}\left(i\right)$ for each segment *i*, and the corresponding difference $\Delta_{3}\left(i\right)=N^{+}\left(i\right)-N^{-}\left(i\right)$; and finally perform a sign test for the differences $\Delta_{3}$, assuming these are independent and equally distributed. While this introduces the need for longer time series and of the definition of an additional parameter (i.e., the segment length), it also simplifies the analysis of time series that are non-stationary, or that are irreversible only a fraction of the time.

### 2.6. Micro-Scale Trends (MSTrends) Test

Also indirectly leveraging the concept of permutation patterns, ref. [99] proposed a test based on micro-scale trends, i.e., the slope of a polynomial fit performed over small overlapping windows of the original time series. The time reversal operation changes the sign of these slopes; thus, if a subwindow is associated with a slope of +α, the corresponding time-reversed subwindow will have a slope of −α. As the probability distribution of these slopes must be the same in the original and time-reversed time series for this to be reversible, the probability distribution must be symmetrical with respect to zero.

As in previous cases, let us start from a time series $\left\{x_{t}\right\}$ composed of *T* observations, which we divide into N−δ+1 overlapping windows of length δ, such that the *i*-th window is defined as $w_{i}=\left\{x_{i},\ldots ,x_{i+\delta -1}\right\}$. Afterwards, a least squares polynomial fit of degree d<δ is applied to each window, and the highest power coefficient *a* is extracted. Finally, two probability distributions of *a*, for the original and time-reversed time series, are extracted and compared through a Kolmogorov-Smirnov test.

Note that this test is conceptually similar to the permutation patterns and the ternary coding ones, as all three are based on detecting correlations in consecutive elements of the time series. The main difference resides in the fact that the micro-scale trends test explicitly includes the amplitude of the signal, or more specifically the magnitude of the changes in the time series, in the assessment of irreversibility.

Finally, as also proposed in ref. [99], this test can be applied to any modification of the original time series. Specifically, suppose that a new time series $y_{t}$ is created from $x_{t}$, by calculating the second (standard deviation) or third (skewness) central moments of the sub-time series $\left\{x_{t},\ldots ,x_{t+\Delta }\right\}$ (note that Δ≠δ, and that usually Δ≫δ). The proposed test can then be applied to the new time series *y*, in order to, for instance, detect time series alternating between upward and downward movements with an increasing (or decreasing) frequency.

### 2.7. Visibility Graphs

One recent and innovative approach to time series analysis is represented by a family of methods that map a time series into the nodes of a network, based on geometric criteria [100,101]. In all of these methods, a complex network [102] is created, whose nodes correspond to the individual data of the time series; pairs of nodes are then connected when they fulfil some geometrical rule, usually based on whether they can “see” each other-hence the name “visibility graph”. Such network is then analysed using standard complex network theory tools, and involves for instance quantifying the degree distribution or other topological metrics or the network induced by the data [103].

Time series irreversibility has been assessed using one such methods, viz. the so-called directed Horizontal Visibility Graphs (dHVG), in which pairs of nodes are connected if the line connecting both values is not obstructed by another intermediate point [101]. In other words, given two nodes *i* and *j*, a link is created if $x_{i},x_{j}>x_{n},\;\forall n|i<n<j$, with $x_{i}$ being the element of the time series mapped into node *i*. Afterwards, the irreversibility of the time series can be assessed by comparing the distributions of in- and out-degrees (i.e., respectively the number of links arriving to and departing from a given node), and by calculating a Kullback-Leibler divergence. Specifically, as the in-degree of a node becomes its out-degree under a time reversal transformation, reversibility implies equality of both distributions and a Kullback-Leibler divergence converging to zero [51,104].

**Figure 1:**
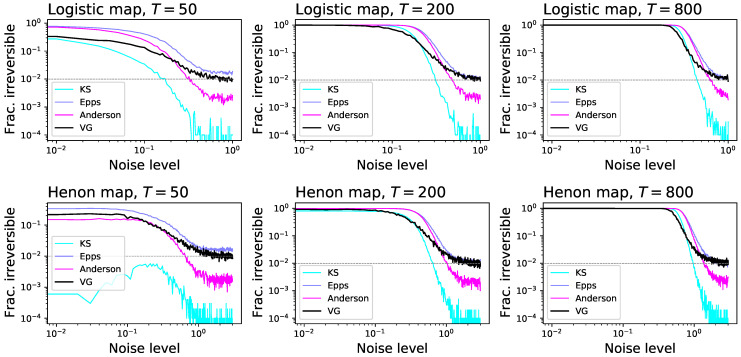
Performance of different statistical tests applied to the Visibility graph methodology. Each panel reports the evolution of the fraction of time series identified by the test as irreversible, as a function of the noise amplitude. Top panels correspond to time series generated through the logistic map, bottom ones through the Henon map - see Section 3.2 for definitions. Left, central and right panels respectively correspond to time series lengths of T=50, 200 and 800. The horizontal dashed line in each panel represents the expected fraction of time series detected as irreversible for a significance level α=0.01.

Note that the original study using dHVG to assess irreversibility only proposes the use of the Kullback-Leibler divergence as a metric of the amount of irreversibility, but that this is not a statistical test, and as such cannot yield a *p*-value. This shortcoming can be solved, on one hand, by comparing the obtained divergence with what observed in surrogate time series [58], albeit at a large computational cost. On the other hand, one can use standard statistical tests to assess the equality of the two in- and out-degree distributions. In order to test this option, we report in Figure 1 the fraction of times an irreversible time series is detected as such by the visibility graph method with $10^{4}$ surrogates (black lines), by a Kolmogorov-Smirnov (KS) test (cyan lines), by an Epps-Singleton test (blue lines) [105], and an k-sample Anderson-Darling test (magenta lines) [106]. Time series were generated with two classical chaotic maps, specifically the logistic and the Henon map (see Section 3.2 for definitions), for T=50, 200 and 800, and with different amplitudes of a Gaussian additive noise. It can be appreciated that the Epps-Singleton test outperforms the surrogate approach, being more sensitive for short time series and high levels of additive noise. It is also more sensitive than the KS test, whose use was previously proposed in [107]; this is easy to explain, as the Epps-Singleton test is specifically designed to test data coming from discrete distributions, as is the case of the degree, while the KS test expects continuous ones.

### 2.8. Local Clustering Coefficient

Based on the previous concept of visibility graph, Donges and coworkers proposed to assess irreversibility not just by looking at differences in the degree distributions, but more generally, in any topological property of the obtained visibility networks [107]. They specifically focussed on the local clustering coefficient, a measure assessing the degree to which nodes in a graph tend to form triangles. Two versions of the metric are created, respectively describing the connectivity due to past and future observations, and thus resulting in the *retarded* and *advanced* local clustering coefficients:

(8) Cir=kir2−1∑j<i,k<iai,jaj,kak,i

and

(9) Cia=kia2−1∑j>i,k>iai,jaj,kak,i.

As standard in complex network theory, $a_{i,j}$ represents the element (i,j) of the adjacency matrix A, and its value is one if a link exists between nodes *i* and *j*, and zero otherwise. Additionally, $k_{i}^{r}$ and $k_{i}^{a}$, respectively, represent the retarded and advanced degrees of node *i*, i.e., its number of connections with previous and following nodes. Once the two distributions $C_{i}^{r}$ and $C_{i}^{a}$ have been obtained, these have to be compared. As in the case of the visibility graph, this can be done through a Kullback-Leibler distance, using surrogate time series to extract a *p*-value; or through statistical tests, like the KS or the Epps-Singleton [105] ones.

### 2.9. Additional Irreversibility Tests

For the sake of completeness, we also mention a few additional irreversibility tests that have been proposed in the literature. These were not included in the analysis presented below due to a number of reasons, including their computational cost that makes them unsuitable for most real-world problems; their problem-specificity narrowing their scope; or their overlap with other included methods.

This list should indeed start with the test proposed by Yves Pomeau in 1982 [5], to the best of our knowledge the first instance of an irreversibility test, and which probably received way less attention than deserved for being written in French. Pomeau proposed to use any function asymmetric with respect to time, as for instance

(10) $$\psi \left(\tau \right)=\bar{x\left(t\right)\left[x(t+2\tau )-x(t+\tau )\right]x\left(t+3\tau \right)},$$

being τ a lag constant. The idea proposed in ref. [5] is important for introducing several relevant concepts: the fact that multiple functions, similar to ψ, could be introduced, i.e., the idea that no single definitive solution to the irreversibility problem exists; and the introduction of a lag τ, effectively enabling the possibility of multi-scale analyses. On the other hand, ψ is only a metric of irreversibility; in order to create a statistical test, the function has to be calculated over a large number of surrogate time series, with the consequent major computational cost. Finally, the attentive reader will easily recognise the similarity between this and the Ramsey test of Section 2.2.

Following a chronological order, the next test worth mentioning is the one proposed by Diks and coauthors [108]. It is based on a test checking whether two sets of vectors are drawn from the same multi-dimensional probability distribution; specifically, the distance between pairs of vectors is calculated, for then deriving an unbiased estimator and the expected variance under the null hypothesis of independence. Given a time series $\left\{x_{t}\right\}$, its irreversibility can be estimated by comparing vectors representing *x* and its time-reversed version; these vectors can easily be created by considering non-overlapping sub-windows with embedding dimension *m*, and eventually an embedding delay τ, as customary in permutation pattern analyses [94,95].

Costa, Goldberger and Peng proposed a method to study the irreversibility of heartbeat dynamics, based on the analysis of the increases and decreases in heart rate [62]. A heart rate time series $X=\{x_{i}\}$ is converted into a sequence of heart rate increments and decrements $Y=\{y_{i}\}$, $y_{i}=x_{i+1}-x_{i}$, representing the competition between the neuroautonomic stimuli controlling the heartbeat. Such increments and decrements are then studied from a thermodynamical perspective, the authors specifically supposing that they require an amount of energy related to their magnitude. If the time series is reversible, then the average energy for heart rate “activation” (i.e., for $y_{i}>0$) must be similar to the average energy for its “relaxation” ($y_{i}<0$); hence a test can be constructed by calculating the difference between the two average energies, and comparing the result with the one obtained for surrogate time series. The approach is further generalized by proposing a time asymmetry index $\hat{A}$, defined as:

(11) $$\hat{A}\left(\tau \right)=\frac{\sum_{y_{\tau }>0}p\left(y_{\tau }\right)\ln p\left(y_{\tau }\right)}{\sum_{y_{\tau }}p\left(y_{\tau }\right)\ln p\left(y_{\tau }\right)}-\frac{\sum_{y_{\tau }<0}p\left(y_{\tau }\right)\ln p\left(y_{\tau }\right)}{\sum_{y_{\tau }}p\left(y_{\tau }\right)\ln p\left(y_{\tau }\right)},$$

with $y_{\tau }$ being the average of *y* inside moving windows of size τ, thus introducing the possibility of a multi-scale irreversibility; and $p(y_{\tau })$ representing the probability of finding a discrete value $y_{\tau }$. Note that this index $\hat{A}$ is assessing the difference between the probability distributions of both increase and decrease trends in the time series, and is thus generalised by the MS Trends test for δ=2 and d=1.

An even simpler version of the time asymmetry index $\hat{A}$ of Equation (11) proposed by Wang, Shang and Fang involves comparing the number of times a time series increases or decreases [109]:

(12) $$\hat{A}\left(\tau \right)=\frac{\sum \Theta \left(-y_{\tau }\right)-\sum \Theta \left(y_{\tau }\right)}{N-\tau },$$

Θ being the Heaviside function, and *N* the number of elements composing the original time series *X*. This metric thus simply counts the difference between the number of increase and decrease trends, and as such is included in the previously described MS Trends. This metric is also conceptually close to Porta’s [64] and Guzik’s [67] indices-see also ref. [69] for a method based on these two.

A completely different approach has more recently been proposed by Alvarez-Ramirez and co-workers [110], based on a modification of the concept of multi-fractal detrended fluctuation analysis (DFA) [111]. The analysis starts like a standard DFA, i.e., the time series is divided in sub-windows of different sizes, and a detrending process is executed on each one of them. The average fluctuation function is then calculated on these sub-windows, but discriminating those windows according to the direction of their main slope, i.e., increasing and decreasing. As sub-windows with upward trends become downwards under the operation of time reversal, the irreversibility of the time series can be estimated through the distance between the two (upward and downward) fluctuation functions.

The Visibility Graph approach presented in Section 2.7 has been modified by several authors. Some proposals include the use of an asynchronicity metric to compare the sequences of degrees of nodes, in the forward and backward time series [112]; the use of permutation patterns to encode node degrees [113]; the use of higher moments [114] and singular value decomposition [115] to analyse the node degree sequence; the analysis of motifs [116]; and ways to encode multivariate time series in the visibility graph [117].

Salgado-García and Maldonado recently proposed a method for estimating the entropy rate and the entropy production rate associated with a (finite and symbolic) time series [118], based on estimating three recurrence-time statistics, namely the return time, the waiting time, and the hitting time. If the underlying dynamics is time-irreversible, then the entropy production rate for the forward and backward time series should be different; a test can then be constructed, by estimating the two rates, and check whether they are different in a statistically significant way. It is nevertheless worth noting that the estimation of these recurrence-time statistics requires long time series, as e.g., authors applied the method to time series generated with chaotic maps of $8\times 10^{6}$ points of length [118]; the applicability of this test to real-world time series may thus be challenging.

Finally, Dabelow, Bo and Eichhorn consider the problem of studying the trajectory of a Brownian particle in a thermal bath, also subject to active processes like self-propulsion or collisions with other active particles [119]. They show that this results in a time irreversibility, as the probabilities of trajectories forward and backward in time diverge in a way described by a non-local memory kernel. Most importantly, this irreversibility can be estimated from the trajectories themselves-see Equation (1b) in ref. [119].

## 3. Evaluation and Comparison

### 3.1. Preliminaries

As customary, we here evaluate each irreversibility test by generating a large amount of time series, using different dynamical systems and maps of known irreversibility, and by comparing the fraction of times each test correctly identifies their nature. Before presenting the obtained results, an important issue has to be clarified. Almost all tests here considered include some parameters that have to be specified. As will be discussed below, the values of these parameters have a substantial impact in the results, but no guidelines or rules are available to define them. As a (admittedly suboptimal) solution, we here test multiple possibilities (see Table 1 for a full list), for then keeping the one yielding the lowest *p*-value. In order to correct for multiple comparisons, this latter *p*-value is finally multiplied by the number of tests performed, thus effectively applying a Bonferroni correction.

### 3.2. Tests’ Performance on Synthetic Data

**Figure 2:**
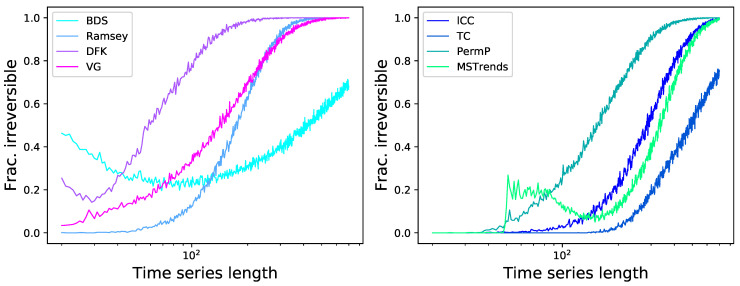
Evolution of the fraction of time series detected as irreversible by the eight considered tests, as a function of the time series length. Time series have been created according to the noisy logistic model and with a noise level of σ=0.2, see main text for details. Reported values correspond to the average over $10^{3}$ realisations. The eight methods are distributed in two panels for the sake of clarity.

**Figure 3:**
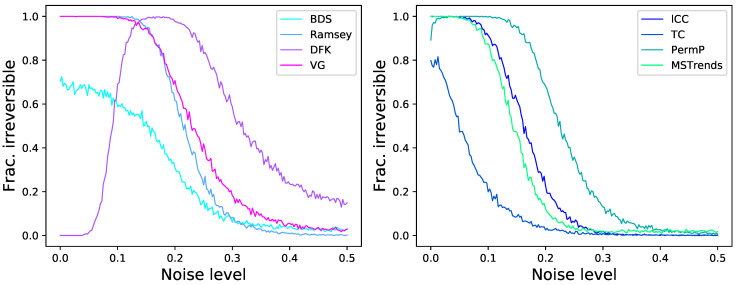
Evolution of the fraction of time series detected as irreversible by the eight considered tests, as a function of the noise level σ. Time series have been created according to the noisy logistic model and with a fixed length of T=200, see main text for details. Reported values correspond to the average over $10^{3}$ realisations.

As a first simple approach to the comparison of the eight tests previously described, we start by considering time series generated by the well-known logistic map, defined as $x_{n+1}=ax_{n}\left(1-x_{n}\right)$, with a=4.0. This is an example of a chaotic and dissipative map, and is by definition irreversible [120]; additionally, due to the clear structures it induces in the resulting time series *x*, its irreversibility should be easy to detect even in short time series, as is its chaotic nature. We further consider the case of an additive observational noise, such that the analysed time series are given by $y_{n}=x_{n}+N\left(0,\sigma \right)$, with N being random number drawn from a normal distribution of zero mean and σ standard deviation. We here report two results. First, Figure 2 reports the fraction of time series that are detected as irreversible by each method, as a function of their length and for a constant noise level of σ=0.2; secondly, Figure 3 reports the same fraction as a function of the noise level, for a fixed length of T=200. While all methods tend to recognise all time series as irreversible, provided enough data are available and noise is low enough, some interesting behaviours can be appreciated. First of all, the eight methods are quite heterogeneous in their performance, with minimum lengths for reaching a perfect identification going from ≈200 for DFK to almost 2000 for BDS. Also, BDS seems to be slightly more effective with short (i.e., T<50) time series than with intermediate ones-even though this is due to a larger probability of getting false positive results, as will later be discussed. Finally, while noise is almost always detrimental to the correct identification of the irreversibility nature of the time series, as uncorrelated noise is by definition reversible; yet, and most surprisingly, a small amount of noise is essential in the case of DFK.

**Figure 4:**
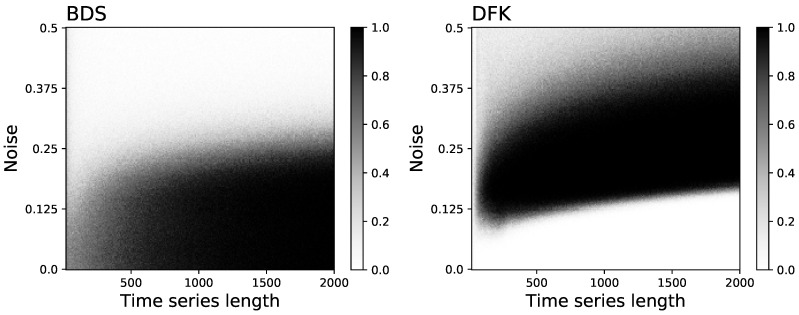
Evolution of the fraction of time series detected as irreversible by the BDS (left panel) and DFK (right panel) tests, as a function of the time series length (X axes) and the noise level σ (Y axes). Time series have been created according to the noisy logistic model described in the main text. Each point represents the result for 200 independent time series.

In order to better understand the behaviour of the BDS and DFK tests, Figure 4 reports the evolution of the fraction of time series detected as irreversible as a function of both the time series length and the noise level. Most interesting, the longer are the time series, the higher is the minimum level of noise required by the DFK test.

We then move to a more complete comparison, using the following four well-known and irreversible dynamical systems:

- the previously defined logistic map $x_{n+1}=ax_{n}\left(1-x_{n}\right)$, with a=4.0, and without observational noise;
- the Henon map, defined as $x_{n+1}=1+y_{n}-ax_{n}^{2}$, $y_{n+1}=bx_{n}$, with a=1.4 and b=0.3;
- the Generalised Autoregressive Conditional Heteroskedasticity (GARCH) model [85], defined as $x_{t}=\sigma_{t}z_{t}$, with $\sigma_{t}^{2}=\alpha^{*}\left(1+\sum_{i=1}^{3}2^{-i}\right)x_{t-i}^{2}+\sum_{i=1}^{3}2^{-j}\sigma_{t-j}^{2}$, $\alpha^{*}=1.2$, and $z_{t}$ being independent random numbers drawn from an uniform distribution U(0,1); and
- the three variables of the Lorenz chaotic system, a continuos system defined as $x^{\prime }=\sigma \left(y-x\right)$, $y^{\prime }=x\left(\rho -z\right)-y$, and $z^{\prime }=xy-\beta z$, with ρ=28, σ=10 and β=8/3.

**Figure 5:**
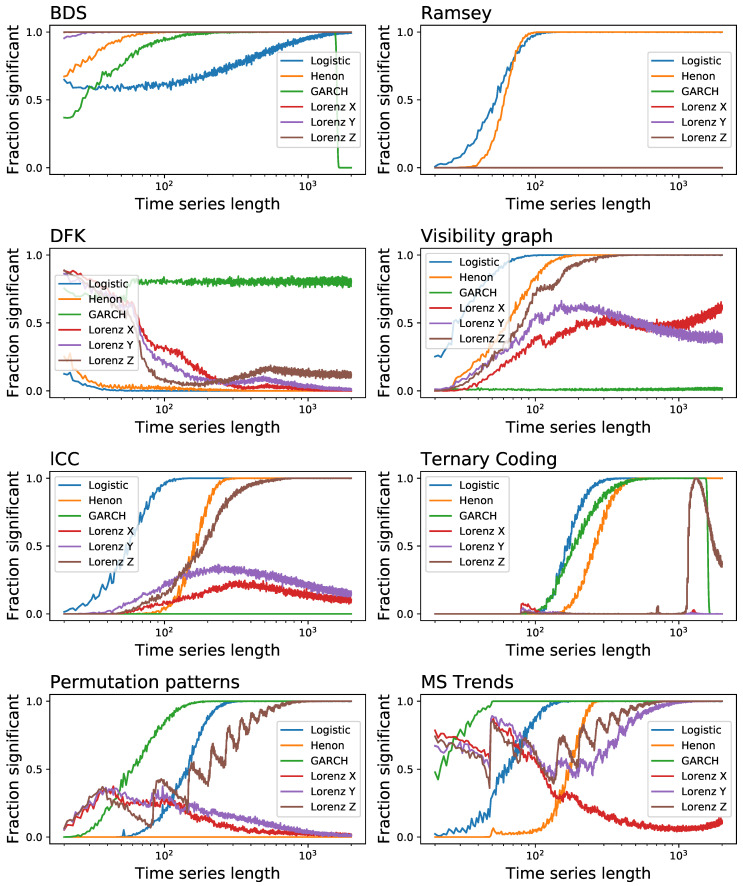
Evolution of the fraction of time series detected as irreversible by the eight tests here considered, as a function of their length. Each point corresponds to the execution of $10^{3}$ random realisations. See main text for definitions of the dynamical systems.

The results, reported as the fraction of time series that are detected as irreversible as a function of their length, are depicted in Figure 5. The global picture is one of great heterogeneity, with no common patterns between different tests, and especially with no clear winner. While the BDS test is generally outperforming all the other ones, it yields substantially inferior results in the case of the logistic map-a quite surprising result, considering the clear irreversible nature of its time series. On the other side, it is easier to find which tests underperform: the Ramsey, DFK and Ternary Coding might be avoided. Note that the Ramsey test was found to be more sensitive than the BDS one [89]; yet, this was based on the study of Self-Exciting Threshold AutoRegressive (SETAR) models alone, without any confirmation with other tests. As a final note, it is worth noting the case of DFK, which seem to perform much better in the case of short time series-and it actually outperform most other tests in the case of the Lorenz system, for time series with less than 50 observations.

**Figure 6:**
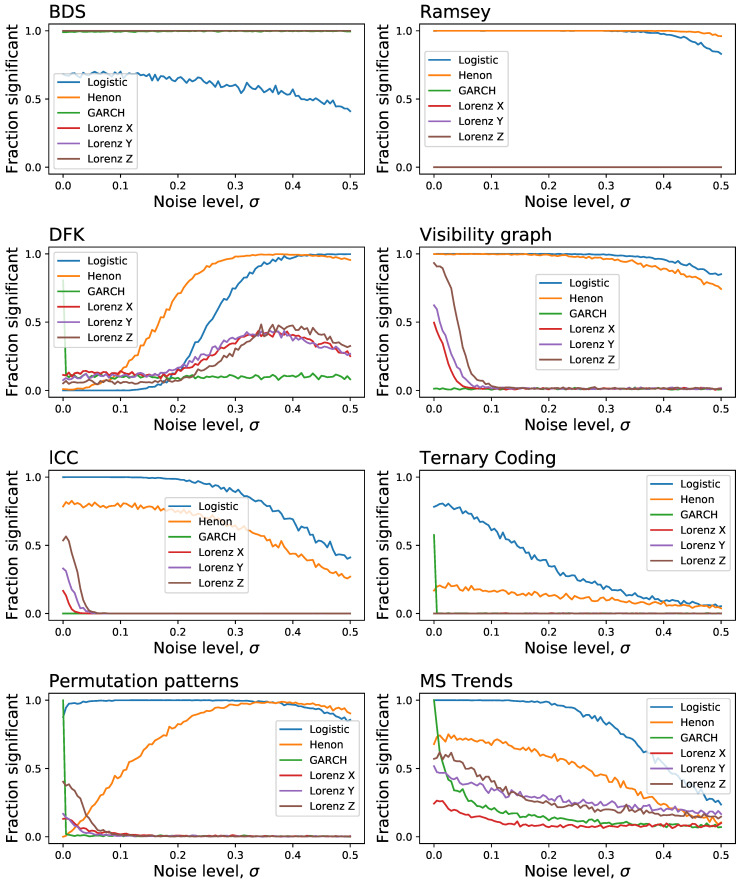
Evolution of the fraction of time series detected as irreversible by the eight tests here considered, as a function of the observational noise level, and for a fixed length of T=200. Each point corresponds to the execution of $10^{3}$ random realisations. See main text for definitions of the dynamical systems.

In a way similar to Figure 3, we report in Figure 6 the evolution of the fraction of time series detected as irreversible by each method as a function of the observational noise level added to them, and for a fixed length of T=200. The noise is defined as random numbers drawn from a N(0,σ) distribution, which are then multiplied by the standard deviation of the time series *x* in order to maintain a constant relative amplitude; in other words, σ=0.5 represents a noise with half the standard deviation of the original time series. On one hand, and as expected, observational noise is hindering the ability of the tests to detect irreversibility, although at different levels-for instance, BDS seems very resilient to noise. On the other hand, one can again see that some levels of noise can be beneficial, as is the case of DFK (for almost all dynamical systems) and Permutation Patterns (for the Henon map).

We also analyse one aspect that is usually overlooked in works proposing new irreversibility tests, i.e., the probability of finding false positives, or when the tests detects as irreversible a time series that is actually reversible. For that we considered the four reversible systems:

- time series composed of random number extracted from a normal distribution N(0,1);
- time series composed of random number extracted from a uniform distribution U(−1,1);
- the *x* variable of the Arnold Cat map, a conservative (and hence reversible) chaotic map defined as: $x_{n+1}=\left(x_{n}+y_{n}\right)mod\left(1\right)$, $y_{n+1}=\left(x_{n}+2y_{n}\right)mod\left(1\right)$.
- the Ornstein-Uhlenbeck process, i.e., a mean-reverting linear Gaussian process *T* [1].

**Figure 7:**
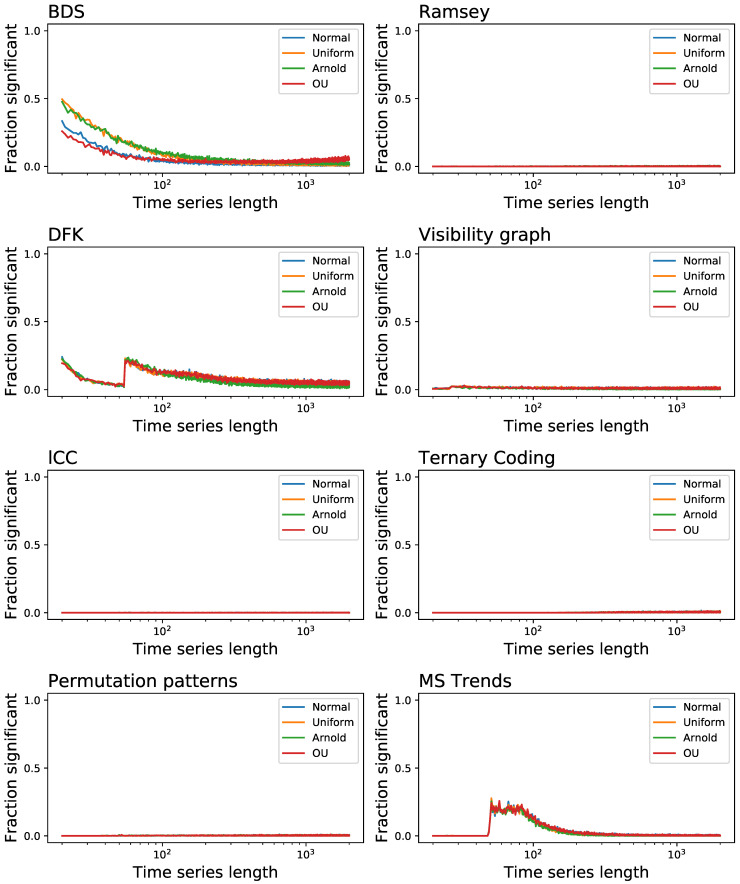
Evolution of the fraction of time series detected as irreversible for series generated by four reversible systems, as a function of their length. Each point corresponds to the execution of $10^{3}$ random realisations. See main text for definitions of the dynamical systems.

Figure 7 reports the fraction of time series that are identified as irreversible, as a function of their length *T*. Note that the significance level for rejecting the null hypothesis of reversibility has been set to α=0.01; hence, a 1% error rate is to be expected and does not imply a bias in the results. Most of the tests behave extremely well, with the exception of BDS, DFK and MS Trends, which yield a larger than expected number of false positives, especially for short time series. This also explains the results for the BDS in Figure 2: the high fraction of short time series detected as irreversible is partly the result of a general overestimation of the irreversibility, which also produces undesirable false positives.

**Figure 8:**
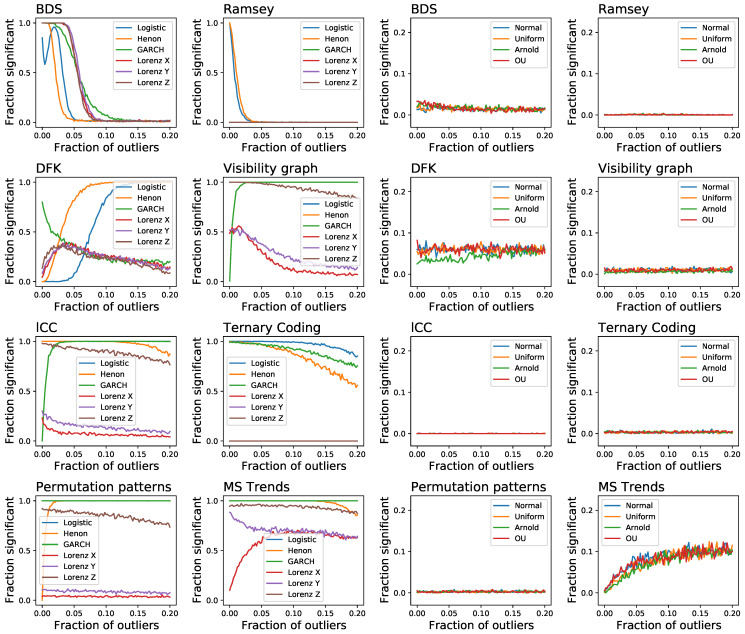
Evolution of the fraction of time series detected as irreversible by the eight tests here considered, as a function of the fraction of outlier events, and for a fixed length of T=200. Each point corresponds to the execution of $10^{3}$ random realisations. Note that the two leftmost columns correspond to irreversible dynamical systems, while the two on the right to reversible ones.

As a final issue in this section, we tackle yet another aspect that is usually not considered in the analysis of irreversibility tests, specifically their resilience to the presence of extreme values. This is achieved by generating time series with all previously described dynamical systems, both reversible and irreversible, and by adding a fraction of extreme events, these being defined as random numbers drawn from a U(−10,10) distribution, which are then multiplied by the standard deviation of the time series in order to maintain a constant relative amplitude. Note that this is similar, but not the same as adding noise. To illustrate, consider a short random time series *x*, of e.g., 20 observations, with values drawn from N(0,σ); if two extreme values $x_{5}=10$ and $x_{15}=20$ are added, these create an asymmetry in time and hence make the time series irreversible. Results of Figure 8 suggest some interesting behaviours. On one hand, tests like BDS and Ramsey, that were quite resilient to random noise, loose their sensitivity even for small fractions of outliers; on the other hand, DFK and MSTrends yield a high number of false positives. Additionally, we again see the beneficial effect that this kind of noise has on some tests, e.g., DFK, lCC and even MSTrends, in the identification of some irreversible dynamical systems.

### 3.3. Ensemble Testing

Given the previous results, one may ask whether it is possible to define similarities and complementarities between the different tests; and, in the latter case, whether complementary methods could be combined to create an “ensemble test”, as commonly done in machine learning [121]. This task is nevertheless not easy, as the performances of tests are quite heterogeneous, as seen in Figure 5 and Figure 6. As a solution, we here created a set of $10^{4}$ time series of random type, i.e., each time series if generated from six types of Figure 5 with equal probability; of random length, drawn from a uniform distribution between 50 and 2000; and with a random level of observational noise, drawn uniformly from σ=0 to σ=0.5. Each time series is then analysed by the eight considered tests. In short, this allows us to compare the behaviour of the tests using a set of time series covering a wide array of types and parameters.

**Figure 9:**
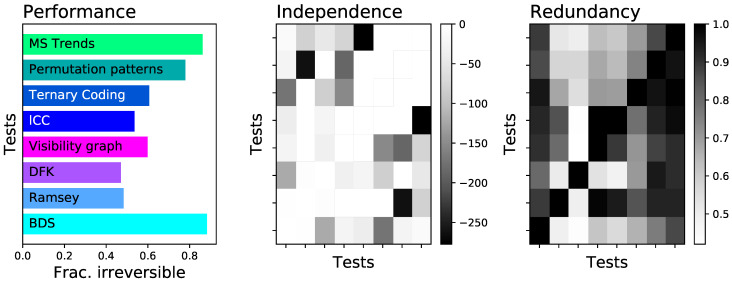
Performance of tests on a set of mixed time series. (**Left**) Fraction of time series detected as irreversible by each test. (**Centre**) $\log_{10}$ of the *p*-value of a Chi-square test of independence between the results of pairs of tests. (**Right**) Pairwise redundancy between tests, measured as the fraction of time series detected as irreversible by the test in the column that are also detected as such by the test in the row. In the central and right panels, tests are sorted from left to right and from top to bottom, following the same sequence as in the left panel; the BDS test is thus always located in the bottom left corner, the MS Trends one in the top right.

The left panel of Figure 9 firstly reports the fraction of time series detected as irreversible by each algorithm. As may be expected from Figure 5, a great variability is present, with performances spanning from 0.5 to 0.9. Note that tests like VG and lCC, which performed well in Figure 5, here suffer from the presence of observational noise-their sensitivity to noise was depicted in Figure 6. On the other hand, the two best tests seem to be BDS and MSTrends, with fractions of detected time series of, respectively, 0.88 and 0.86.

The use of a common set of heterogeneous time series also allows for two additional analyses. Firstly, the central panel of Figure 9 reports the $\log_{10}$ of the *p*-value of a Chi-square test of independence between the results of pairs of tests. In other words, the larger the *p*-value (i.e., the smaller the $\log_{10}$, in absolute value, and the lighter the color of the corresponding cell), the more independent are the results yielded by the two tests under analysis. Secondly, the right panel of Figure 9 reports the pairwise redundancy between tests, i.e., the fraction of time series detected as irreversible by the first test that are also detected as such by the second one.

These results suggest four possible types of ensemble testing:

- Combining all eight tests into a single one (here denoted by *Ens:all*).
- Combine the two most effective tests, i.e., BDS and MSTrends (*Ens:BDS_MSTrends*).
- Combine the pairs of tests that yield the most independent results, such that the errors of one of them could be corrected by the other. These pairs would be Ramsey and PermP (*Ens:Ramsey_PermP*), and lCC and MSTrends (*Ens:lCC_MSTrends*).

In each case, the resulting *p*-value would be the minimum among the *p*-values yielded by the merged methods, multiplied by the number of tests, in order to correct for multiple testing. When these four ensemble tests are applied to the time series composing the previously described set, they yield fractions of irreversible time series of 0.88 (*Ens:all*), 0.97 (*Ens:BDS_MSTrends*), 0.81 (*Ens:Ramsey_PermP*), and 0.86 (*Ens:lCC_MSTrends*). Combining different tests can thus yield better results, as the shortcomings of one can be countered by the other ones. Still, the necessary condition is that each test should be good enough, as the complementarity alone is not sufficient. This is clear in the case of *Ens:all*: adding many underperforming tests is not necessarily beneficials, as they do not contribute to the final result, but instead increase the necessary correction for multiple comparisons.

**Figure 10:**
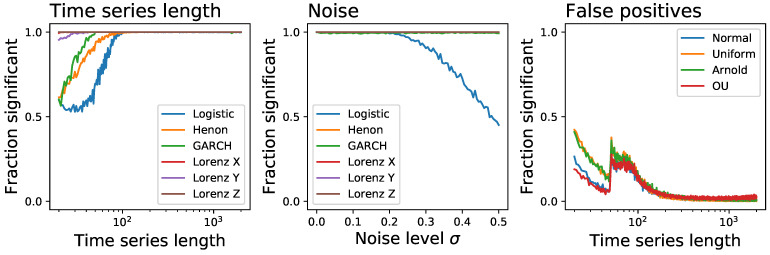
Performance of the ensemble test *Ens:BDS_MSTrends*, combining the BDS and MSTrends tests. (**Left**) Fraction of time series detected by *Ens:BDS_MSTrends* as irreversible as a function of their length. (**Centre**) Fraction of time series detected as irreversible as a function of the noise level σ, for a fixed length of T=200. (**Right**) Fraction of time series detected as irreversible, as a function of their length, for four dynamical system that are not irreversible-thus representing the probability of obtaining false positives.

The performance of the best ensemble test, i.e., combining BDS and MSTrends, is represented in Figure 10; specifically, the three panels depict the evolution of the fraction of time series detected as irreversible as a function of their length (left panel), as a function of the observational noise to them added (center), and the evolution of the fraction of false positives (right). The resulting test behaves in an excellent way for time series longer than T=100, independently on the underlying dynamical system, and also for relatively high levels of observational noise. The only drawback is a significant number of false positives when T<100, region of the parameter space that ought to be avoided.

### 3.4. Analysing Real-World Data: The Case of Human Electro-Encephalography

If the analysis of synthetic data depicted a complex scenario, things only get more interesting and complex when moving to real-world data. As a prototypical example, we here present an irreversibility analysis of human electro-encephalography (EEG) data representing the brain activity of control subjects and patients suffering from alcoholism [122,123,124] and freely available at https://archive.ics.uci.edu/ml/datasets/EEG+Database, accessed on 4 November 2021. Each recording corresponds to the execution of a standard object recognition task [125], and includes 64 time series (i.e., one for each of the 64 electrodes or channels) of 256 elements (i.e., one second of brain activity recorded at 256 Hz). Note that the assessment of the irreversibility on this data set is a challenging task, as, firstly, time series are intrinsically noisy due to the technical limitations of EEG recordings; and secondly, they are short, following brain non-stationarity [124]. On the other hand, a large number of trials are available in both groups (respectively 2010 and 3467 for control subjects and patients), thus enabling robust statistical results.

**Figure 11:**
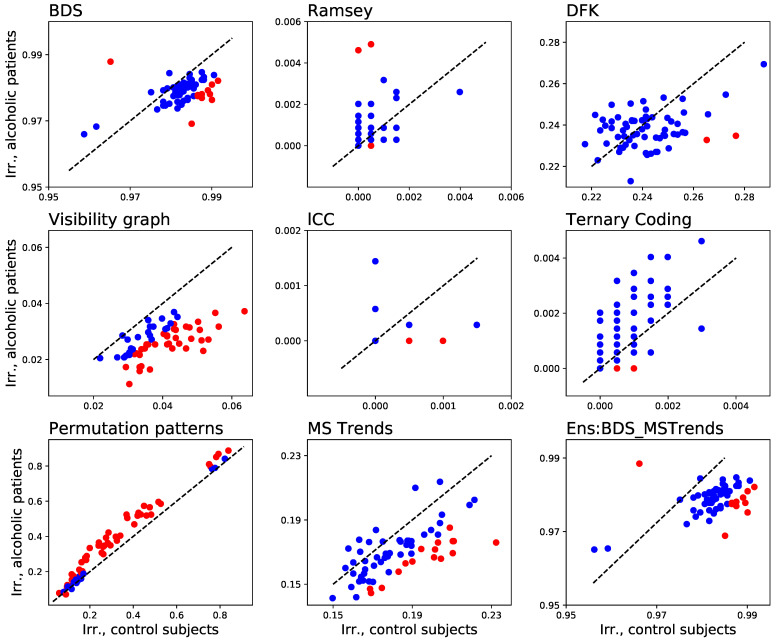
Analysis of human EEG time series. Each panel reports, for a given irreversibility test, the fraction of time series corresponding to alcoholic patients detected as irreversible, as a function of the fraction corresponding to control subjects. Each point represents a single EEG channel, with channels having a statistically significant difference between both groups marked in red.

In Figure 11 we report the results obtained with nine irreversibility tests, i.e., the eight here reviewed, plus the ensemble BDS_MSTrends test proposed above. Each panel is a scatter plot of the fraction of time series of patients detected as irreversible, as a function of the fraction for control subjects. Each of the 64 points in each graph corresponds to one of the EEG channels (or electrodes, or sensors); points are further coloured in red if a statistically significant difference between the two groups is present (according to a binomial test and for α=0.01), and in blue otherwise.

Several interesting conclusions can be drawn. First of all, brain dynamics seems to be highly irreversible, as some tests (BDS and Ens:BDS_MSTrends) can detect irreversibility in up to 99% of the time series; nevertheless, not all tests agree, with some of them (i.e., Ramsey, lCC and Ternary Coding) notably yielding the opposite result. Even more interesting is the behaviour of the permutation patterns test, which detects strong irreversibility only in some EEG channels-on the contrary, all other tests seem to find homogeneous results across all channels.

As a second point, a higher sensitivity to irreversibility does not imply a higher sensitivity to differences across groups, and hence a higher usefulness in the analysis task. Specifically, it can be noted in Figure 11 that BDS yields the larger fraction of time series tagged as irreversible; but only a few channels are different between control subjects and patients in a statistically significant way. On the other extreme one can find the VG test, with most of the channels being significantly different, but with only ≈4% of time series being irreversible. To illustrate this, imagine the case of an extremely sensitive irreversibility test, able to detect irreversibility in all time series, i.e., both of control subjects and of patients. All EEG recordings would be classified as irreversible, and it would thus be impossible to discriminate between the two groups.

As a third and final point, the differences between control subjects and patients manifest in different ways depending on the used test. To illustrate, patients are less irreversible according to the VG and MS Trends tests; but are more irreversible according to the permutation patterns one. MS Trends also yields different results depending on the window length considered, and for w=200 it detects both increases and decreases of irreversibility, depending on the EEG channels [99].

**Figure 12:**
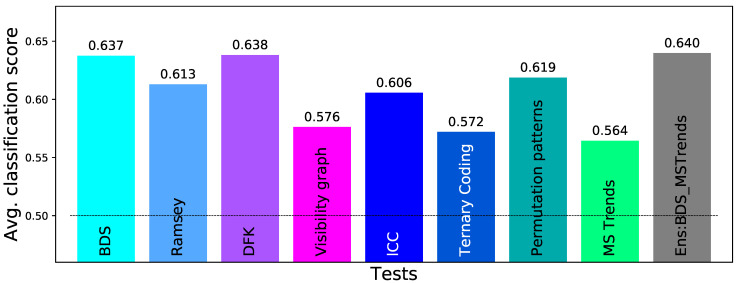
Classification score obtained in the task of classifying EEG recordings according to the corresponding group (control subjects vs. patients), by using the output (i.e., the *p*-value) of each irreversibility test as feature. See main text for details.

Obtaining statistically significant results is nevertheless not the final goal of most real-world analyses; to illustrate, in the case of the data set here considered, a standard problem would be to create a classification model, able to predict, from an EEG recording, whether a new subject is healthy or is instead suffering from alcoholism. In order to simulate such analysis, we have here performed a simple classification task, in which $10^{3}$ trials of control subjects and $10^{3}$ trials of patients are randomly selected; the 64 time series of each trial are then tested for irreversibility, and the resulting *p*-values are fed into a Random Forest machine learning classifier [126] (with a maximum depth of 4 and 2000 estimators, using the Python implementation included in Scikit-learn [127]). In order to avoid overfitting, a 10-fold cross-validation has been executed; and the whole classification process has been repeated 20 times, for obtaining an average classification score. The results, represented in Figure 12, add yet another dimension. Specifically, Ramsey, i.e., a test not standing out in Figure 9 and Figure 11, here yields results comparable to the best tests; MS Trends, on the other hand, here yields the worst classification score. It is thus clear that obtaining statistically significant differences between two groups does not imply yielding a large quantity of information about such differences. This also highlights that choosing the best irreversibility test for a given real-world application is far from being a simple problem.

### 3.5. Computational Cost

**Figure 13:**
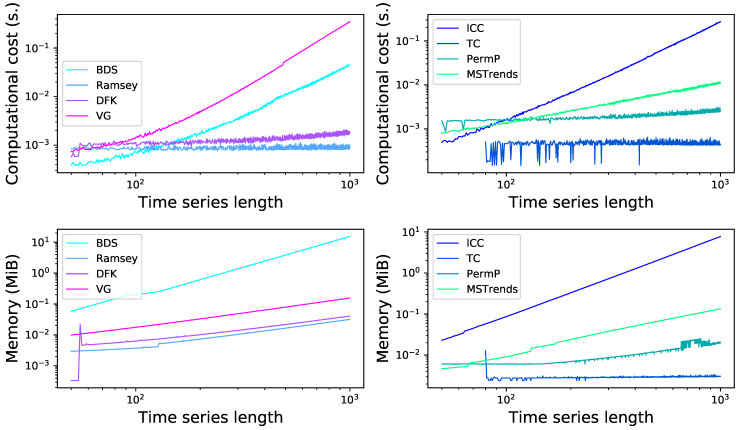
Computational cost (**top**) and memory requirement (**bottom**) of the eight irreversibility tests as a function of the length of the analysed time series. Values are averages, in seconds and in MiB, over 50 realisations using an hex-core Intel Core i5 at 3.3 GHz and a single core; the memory requirement has been measured using the *tracemalloc* Python library.

One final important aspect to take into account in the real-world application of these irreversibility tests is their computational cost. Specifically, Figure 13 reports the evolution of the time to execute these tests in seconds (top panels), and of the allocated memory (bottom panels), calculated on an Apple iMac with an hex-core Intel Core i5 at 3.3 GHz and for a single core, as a function of the time series length. It can be appreciated that some of them scale as a power of the length (note the log-log scale), and specifically that the worst tests are the Visibility Graph, the local Clustering Coefficient and the BDS tests, both in terms of computational cost and memory. This is not surprising, as for instance the VG method requires evaluating the magnitude of each element against all elements following it, i.e., performing $\propto T^{2}$ operations. This creates a large heterogeneity between methods; for instance, for time series of length $10^{4}$, the cost goes from 7.12 milliseconds for TC to 373 seconds for VG. More relevant is the fact that no clear correlation exists between the computational cost and the effectiveness of each method; to illustrate, while BDS and MSTrends are the best performing tests according to Figure 9 and are also among the most computationally expensive ones, VG and lCC clearly underperform with respect to their cost. In time critical applications, and when long time series have to be analysed, the method based on permutation patterns seems to be the best compromise.

## 4. Software

As a complement to this review, we make public a Python software library with the eight tests described in Section 2.1, Section 2.2, Section 2.3, Section 2.4, Section 2.5, Section 2.6, Section 2.7 and Section 2.8, two additional tests drawn from Section 2.9, plus the ensemble test combining BDS and micro-scale trends described in Section 3.3. The library is freely available at https://gitlab.com/MZanin/irreversibilitytestslibrary, accessed on 4 November 2021. These software implementations have been used to obtain all results included in this review, including the computational costs of Figure 13. Some additional notes and clarifications are included below:

- Tests are organised in independent modules, whose names are reported in Table 2. Each test has a main function, always called *GetPValue*, which returns two results: the *p*-value of the test, and the statistic of the test when available. Besides the time series to be analysed, each test expects a set of parameters, which are also described in Table 2.
- The library only requires standard Python 3.* libraries, including Numpy and Scipy [128]. In addition, some modules use Numba to speed up numerical computation [129]; if this library is not available, the corresponding decorator can be safely commented out.
- Some tests include simplifications in their execution; to illustrate, the permutation patterns algorithm only considers patterns of size 3; and the micro-scale trends one only linear regressions of the data. The reader should nevertheless note that this will be a live library, and that such simplifications may disappear in the future. We therefore recommend to check the online documentation of the project.
- In order to simplify the use of the library, it includes an example program *UsageExample.py*, which creates a time series using a logistic map, and sequentially executes all available tests with different parameters. We recommend the interested reader to start by checking this program.

As a final note, all test implementations have been developed in-house, and as such may contain errors or inefficient code; we welcome readers to send us comments, suggestions and corrections, ideally using the “Issues” feature of GitLab. We also welcome new contributions, like new metrics and tests, provided they have been peer reviewed and are already coded in Python.

## 5. Discussion and Conclusions

In this review, we described the main tests designed to detect the irreversible nature of time series, and evaluated eight of them under different conditions, e.g., for different time series lengths or in the presence of noise and extreme values. We further proposed the possibility of creating ensemble methods, i.e., combining multiple tests together to compensate for individual deficiencies; and made available a Python software library, in order to facilitate future uses and comparisons. There are essentially two approaches to time-reversal symmetry: one in which this is seen a geometric property of a process, typically a stochastic one. The other, in which time-reversal symmetry is related to fundamental physical properties underlying such a process. We discuss our results in terms of these two frameworks.

From the former viewpoint, the main message that emerges from this analysis is that, while the concept of irreversibility is a well-defined physical notion, the same is not true for metrics and tests measuring irreversibility. In other words, given a dynamical system defined through e.g., a discrete or continuous map, it is in principle possible to derive the corresponding entropy production, and hence the irreversibility. On the other hand, if all we know of that system is a time series representing its observable dynamics in a potentially noisy and incomplete way, the irreversibility has to be estimated from it, using one of the metrics and tests here discussed. Yet, the researcher should be careful not to confound both concepts.

Our results also indicate that the metrics and tests here described form a complex landscape. Due to the general nature of the definition of irreversibility, i.e., any statistical difference between the forward and time-reversed versions of the time series, tests have been designed to detect heterogeneous, and in many case not overlapping patterns. As a result, no metric is strictly better than all the other ones; the computational cost is not proportional to the performance; and some tests even benefit from the presence of noise. This last point further exemplifies the distance between “theoretical” and “applied” irreversibility: the theory guarantees that the presence of observational noise implies a reduction of irreversibility [7,8], in spite of what observed by some tests. This also exemplifies the need of a more complete and comprehensive evaluation of any new irreversibility test - in other words, testing using a handful of dynamical systems is not enough.

**Figure 14:**
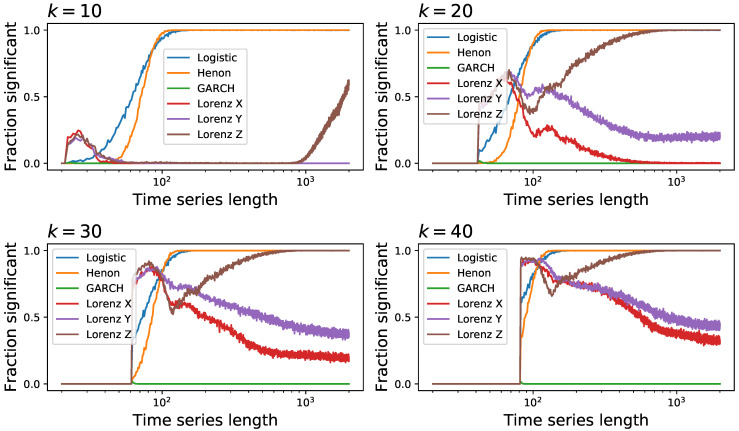
Performance of higher order Ramsey tests. Each panel reports the evolution of the fraction of time series detected as irreversible, as a function of their length, for four maximum values of *k*-see Section 2.2 for definitions.

Many tests include one or more parameters that strongly affect the results. In spite of their importance, there are no guidelines or rules about which values they should take; as a result, the researcher has to test multiple values, with the consequent increase in computational cost-not to mention the risks associated with multiple testing. To illustrate, the Ramsey test has previously been executed with k=(2,3,4); nevertheless, using higher values of *k* improves the result for some types of time series (see Figure 14), as longer correlations can be detected. This is also strongly related to the topic of multi-scale irreversibility, i.e., the fact that time irreversibility may appear only at some specific time scales; while not discussed here, and only seldom considered in real-world studies, this aspect of irreversibility ought to be taken into account in the future.

As a further point, we have seen that real-world analyses may be (and possibly always are) more complex than synthetic case studies. As seen in Figure 11, higher sensitivity is not necessarily a desirable feature when analysing real-world data, especially when the objective is to discriminate between two groups. As well known in machine learning, the objective becomes identifying patterns able to detect differences and enable a classification; whether these patterns correspond or not with a true irreversibility becomes a secondary issue.

What lessons can be drawn from the results here presented? The main one, supporting both optimistic and pessimistic views on the future of time series irreversibility analysis, is that the field has still a long research road ahead. Irreversibility tests are far from being perfect, and most of the tests that have been developed in the last decade are either small modifications of existing ones (see Section 2.9), or do not clearly outperform the first test introduced in 1988 [87,88]. Much additional work is thus needed; yet, what principles should guide this work? We here argue that some guidelines can be obtained by comparing irreversibility analysis with another well-developed type of time series analysis: chaotic tests.

The first difference between time irreversibility and chaoticity testing can be found on the time series used to evaluate the tests. Chaotic maps have a long history, with many of them being proposed and analysed in the last few decades-to illustrate, one may start by checking the long list available in Wikipedia at https://en.wikipedia.org/wiki/List_of_chaotic_maps, accessed on 4 November 2021. This responds to both a theoretical and applied interest, as chaotic maps have found applications in e.g., cryptography [130]. On the other hand, almost no models have been proposed to generate time series with a known time irreversibility, and tests are usually evaluated on time series generated by other systems-most of the time, as in this review, chaotic ones. Two exceptions can be found: Burykin et al. proposed the use of an asymmetric Weierstrass function [131], with a parameter controlling the amount of time irreversibility; and the GARCH model [85], whose parameter α explicitly controls time irreversibility. Yet, we here argue that these models are “too simply irreversible” processes, as the irreversibility can be observed by naked eye, and thus poorly represent real-world scenarios.

The second difference directly stems from the previous point, and can be synthesised in a lack of hard benchmarks for irreversibility tests. To illustrate, chaos tests are usually checked against known chaotic time series [96], with some of them (most notably the *linear congruential generator*) producing time series essentially identical to complete randomness-the linear congruential generator being indeed the algorithm of choice for generating pseudo-random numbers. Having a benchmark system against which most tests fail may generate frustration in researchers; but also offers an excellent way of comparing tests, and more importantly, a reason to keep looking for better solutions. The equivalent of this generator for time irreversibility, i.e., a very challenging generator of time series of known irreversibility, has yet to be proposed-unless, of course, one resorts to the trivial solution of using any irreversible time series and add an extreme amount of observational noise.

One interesting challenge for the scientific community would be to develop a deterministic dynamical system, able to generate time series with tuneable degrees of irreversibility, and whose irreversibility is (i) provable, (ii) not based on observational noise, and (iii) not detected by any of the tests here reviewed.

The complementary approach on time reversal symmetry may provide the conceptual starting point for such an endeavour. From the point of view of the physics underlying an observed process, time-reversal asymmetry is a manifestation of the breaking of detailed balance which characterises various living and physical systems as they operate away from equilibrium. Irreversibility places thermodynamic constraints on important properties of these systems, such as their precision [42], rate [44], speed of change [45], sensing and signalling [132] and predictability [133]. Quantifying the extent to which these systems deviate from equilibrium and produce entropy is therefore key to their characterisation. However, are the existing time-reversal symmetry methods capable of faithfully characterising these properties? Addressing this question involves understanding the limits of time-reversal symmetry methods, in particular when and why these can fail, and the interpretation that one can safely attribute to the results that they yield.

The experimental estimate of time-reversal symmetry is affected by various aspects of available data, viz. the variables used to describe the underlying system, how coarse-grained they are, and the temporal and spatial resolution of the observation. However, for fluctuation theorems relating thermodynamic quantities to hold, all relevant mesostates need to be accessible. Likewise, to assess the entropy production in a stationary process, it is necessary to measure all the currents in the system and their conjugated thermodynamic forces. However, in a typical experimental situation, only partial information about a system is available [41]. Furthermore, defining the degrees of freedom best documenting the non-equilibrium nature of a complex many-body system is in general a non-trivial task [10]. Currently available entropy production estimates from single time series may be inaccurate when only a small set of observables is experimentally accessible. With partial information, distinguishing between forward and backward processes becomes harder. This is because the Kullback-Leibler distance between the forward and backward distributions ρ and $\tilde{\rho }$ decreases when only partial information of the system is used with respect to the distance between ρ and $\tilde{\rho }$ associated with full information [12,18].

Moreover, experimental studies typically analyse coarse-grained versions of the microscopic scales of the system. The identification of thermodynamic quantities at the level of single dynamical trajectories of the stochastic thermodynamic approach relies on time-scale separation between slow degrees of freedom on the one hand, and external time-dependent forces or hidden degrees of freedom on the other hand [40,41,134,135]. When microstates within mesostates, i.e., states in which multiple microstates are lumped together, rapidly thermalise, the entire microscopic structure is recovered at mesoscales. This is not the case when these microstates remain out of equilibrium, as for instance in living systems, leading to additional contributions to the entropy balance [136]. However, while any data with no fine control on microscopic scales miss parts of the genuine physical entropy production of the underlying system, time-reversal symmetry quantifiers may in principle be thought to give a lower bound of the system’s true one in the presence of fast microscopic thermalization [137]. A further aspect that should be taken into account is that, for some systems driven out of equilibrium, broken detailed balance may not be apparent at coarse-grained scales, and irreversibility may sometimes be masked by fluctuations [138,139]. Forward and reverse trajectories may not be asymmetric and currents may vanish even if the underlying system is out of equilibrium [139]. In fact, at large scales, driven systems may even recover thermodynamic equilibrium and obey detailed balance [140]. This aspect makes multiscale measures a necessary tool when analysing complex multiscale systems [62,110]. Overall, while time-reversal symmetry measures can in principle reveal fundamental aspects of the underlying system in a non-invasive way, i.e., without requiring perturbations as is for instance the case with methods assessing the breakdown of fluctuation-dissipation relations, care should be taken not to over-interpret results whether they indicate symmetry or symmetry breaking. Perhaps more direct ways to unveil a system’s thermodynamics will come from methods finding other relations between dynamical and thermodynamical system properties [141,142,143,144,145], possibly resorting to symmetries other than the time-reversal one [44].

## Figures and Tables

**Table 1:** List of parameter values used in the evaluation of each test.

Test	Parameter	Values
BDS	*none*	
Ramsey	*k*	1,2,3,4
DFK	*n*	2,3
	*L*	2,3
Permutation patterns	δ	3
Ternary Coding	*Number of segments*	20
	α	10*^th^* percentile of $\left\{d_{i}\right\}$
MSTrends	δ	2,3,4
	*d*	1
Visibility graph	*none*	
LocalCC	*none*	

**Table 2:** List of tests implemented in the Python software library, with the corresponding parameters and meanings. ${}^{*}$ designates tests that are described in Section 2.9, but not tested in Section 3.

Module (test)	Reference	Parameter	Meaning
BDS	[87,88]	*none*	
Ramsey	[49]	*kappa*	The lag *k*
DFK	[90]	*n*	Number of possible symbols
		*L*	Word length
PermPatterns	[52,84,92,93]	*none*	
TernaryCoding	[98]	*segL*	Length of the segments on which $\Delta_{3}$ is calculated
		*alpha*	Threshold for symbolisation
MSTrends	[99]	*wSize*	Length δ of overlapping windows
		*wSize2*	Length of windows used to calculate higher central moments
VisibilityGraph	[99]	*none*	
LocalCC	[107]	*none*	
Pomeau ${}^{*}$	[5]	*numRnd*	Number of random repetitions to extract the *p*-value
		*tau*	Embedding delay
Diks ${}^{*}$	[108]	*embD*	Embedding dimension, or size of each vector
		*dVar*	Bandwidth of the analysis
Ens_BDS_MSTrends		*none*	

## Data Availability

A publicly available dataset of EEG recordings was analysed in this study, which can be found here: https://archive.ics.uci.edu/ml/datasets/EEG+Database, accessed on 4 November 2021.
